# Overexpression of StBBX14 Enhances Cold Tolerance in Potato

**DOI:** 10.3390/plants14010018

**Published:** 2024-12-25

**Authors:** Heng Zhang, Mingjun Chen, Xiaobo Luo, Li Song, Fei Li

**Affiliations:** 1 Key Laboratory of Plant Resource Conservation and Germplasm Innovation in Mountainous Region (Ministry of Education), Collaborative Innovation Center for Mountain Ecology & Agro-Bioengineering (CICMEAB), College of Life Sciences/Institute of Agro-Bioengineering, Guizhou University, Guiyang 550025, China; hengzzzz2022@163.com; 2 Guizhou Institute of Biotechnology, Guizhou Provincial Academy of Agricultural Sciences, Guiyang 550003, China; 18286033948@163.com (M.C.); xiaoluobopotato@163.com (X.L.); 3 Key Laboratory of Crop Genetic Resources and Germplasm Innovation in Karst Region, Ministry of Agriculture and Rural Affairs, Guiyang 550003, China

**Keywords:** potato, cold resistance, BBX gene, overexpression, transcriptome, WGCNA

## Abstract

Potato (*Solanum tuberosum* L.) is an important food crop, but low temperature affects the potato growth and yield. In this study, the expression level of *StBBX14* was significantly increased over 1 h and then gradually decreased under cold stress. The subcellular localization of the StBBX14 protein took place in the nucleus. The *OE-StBBX14* transgenic lines showed less leaf damage and significantly lower electrolyte leakage compared with the WT under cold stress, indicating that the overexpression of *StBBX14* in the potato enhanced the cold resistance. A transcriptome analysis showed that a total of 2449 and 6274 differentially expressed genes were identified in WT-1 h and WT-12 h, respectively, when compared with WT-0h. A Gene Ontology enrichment analysis revealed that photosynthesis, cell wall, thylakoid, transcription regulator activity, oxidoreductase activity and glucosyltransferase activity were significantly enriched in *OE-StBBX14* and WT. A total of 14 distinct modules were generated by a WGCNA analysis based on all differentially expressed genes (DEGs). Four major modules with cold-related genes were isolated. RT-qPCR analysis showed that the expression patterns of eight DEGs were consistent between the qPCR and RNA-seq. These findings illustrate that the *StBBX14* played an important role in cold stress in potato and provided a data basis for the genetic improvement of cold resistance traits of potato.

## 1. Introduction

Cold stress is one kind of abiotic stress, which can severely impair growth, development, and yield, while also imposing substantial environmental burdens [1]. To withstand these challenges, plants have evolved intricate response mechanisms. Transcription factors (TFs) play a crucial role in regulating the stress response and present promising targets for enhancing crop performance [2]. BBX (B-Box) proteins are a group of zinc-finger transcription factors or regulators with B-box domains or CCT domains [3]. The B-box structural domain in the proteins encoded by the BBX gene family is highly conserved. Although the B-box domains exhibit similar structural and sequence features across different BBX proteins, their functions can vary [4]. The BBX protein family plays a pivotal role in light regulation, encompassing photomorphogenesis, flowering processes, and pigment accumulation [5]. The overexpression of *CmBBX8* promotes flowering under both long- and short-day conditions. UV-B-induced anthocyanin biosynthesis is regulated by *MdBBX22*, which directly interacts with *MdHY5* (*long hypocotyl 5*) and promotes anthocyanin biosynthesis in apple [6]. *VvBBX44* represses the expressions of *VvHY5* and *VvUFGT* (*UDP glucose flavonoid 3-O-glucosyltransferase*) and impedes anthocyanin biosynthesis in grape [3]. *MdBBX37* inhibits anthocyanin biosynthesis in light signaling, which interacts with *MdMYB1* and *MdMYB9* [7]. *PpBBX16* serves as a positive regulator for light-induced anthocyanin accumulation that activates anthocyanin biosynthesis-related genes in pears via *PpHY5* activation [8]. It was demonstrated that *BBX11* loss of function leads to the significant elongation of hypocotyls in mutant seedlings under red-light and long-daylight conditions [9]. Furthermore, *BBX24* functions as a negative regulator by controlling the post-transcriptional activity of *HY5* to regulate photomorphogenesis [10].

Many studies indicate that the BBX gene family is also involved in response to cold stress. The BBX transcription factor *VvZFPL* in grape introduced into *Arabidopsis thaliana* increases the plant’s resistance to low temperatures, high salinity, and drought [11]. Similarly, the expression of *CmBBX24* in *Chrysanthemum* is modulated by *GA4*/*7*, and suppressing this gene reduces the plant’s tolerance to cold and drought [12]. Among various cold-signaling pathways, the C-receptor binding factor (CBF) pathway stands out as the most extensively studied and crucial regulatory mechanism in plants [13]. In apples, *MdBBX37* binds to the promoters of *MdCBF1* and *MdCBF4*, activating their transcription, which also interacts with MdICE1, boosting its ability to enhance the transcription of *MdCBF1* and, in turn, improves the cold tolerance [7]. In tomato, overexpressing *SlBBX17* enhances the cold tolerance mediated by the *CBF* pathway [14]. Additionally, *BBX7* and *BBX8*, which act downstream of *HY5*, are known to positively influence the freezing tolerance by regulating the expression of *COR* genes [15]. It was shown that the loss of *SlBBX31* function correlates with diminished cold tolerance in tomatoes and *SlBBX31* modulates the expression of several ERF transcription factors in response to cold, including *CBF2* and *DREBs* [16].

The potato (*Solanum tuberosum* L.) is one of the four most important food crops in the world. However, existing cultivars of potato prefer cold climates but lack frost tolerance, exhibit sensitivity to low temperatures, and have an inability to acclimate to cold conditions, where even brief exposure to low temperatures can significantly reduce potato yields [17]. Several cold-responsive genes, including *SAD* [18] and *ADC1* [19] were identified and characterized in potato. The overexpression of the *S. commersonii SAD* gene significantly enhanced freeze tolerance in Zhongshu 8 [20]. A previous study demonstrated that the upregulation of *ADC1* expression elevated the putrescine content and conferred improved freezing tolerance in potato [19]. However, the molecular mechanism of cold resistance in potato remains unclear.

In this study, the expression and function of the *StBBX14* gene were analyzed. To further explore the molecular mechanism underlying the roles of *StBBX14* in regulating potato freezing tolerance, wild-type and *OE-*St*BBX14* seedlings were treated with 2 °C for 0 h, 1 h, and 12 h to identify *StBBX14*-*r*egulated COR genes by RNA-sequencing (RNA-seq). The different expression genes in *OE-StBBX14* plants were validated by reverse-transcription quantitative real-time PCR (RT-qPCR) analyses. The findings provide new insights into the molecular mechanisms underlying plant cold tolerance and pave the way for molecular breeding strategies aimed at developing more resilient potato varieties.

## 2. Results

### 2.1. Characterization of BBX14 in Potato Plants

**Figure 1:**
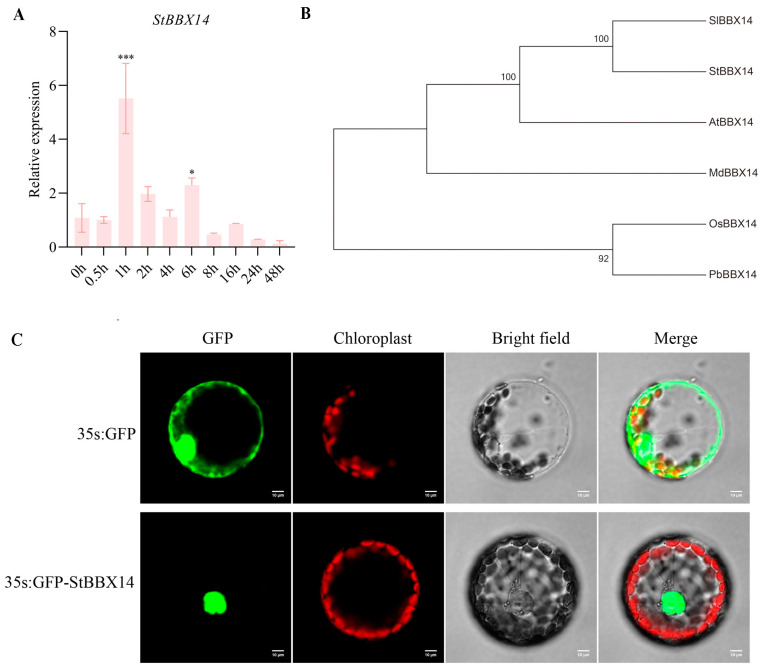
Molecular characterization of *StBBX14*. (**A**) The gene expression of StBBX14 was identified after the 2 °C cold treatment for different times; Student’s *t*-test with * represented *p* < 0.05 and *** represented *p* < 0.001 were used to indicate significant differences. The phylogenetic analysis of the StBBX14 protein. (**B**) The phylogenetic tree was calculated using the maximum-likelihood method by MEGA 7.0 software. Bootstrap values of 1,000 replicates for each branch are shown. The protein sequences from tomato (Sl, *Solanum lycopersicum* L.), *Arabidopsis* (At, *Arabidopsis thaliana*), apple (Md, M*alus domestica*), rice (Os, *Oryza sativa* L.), and pear (Pb, *Pyrus bretschneideri*). (**C**) Subcellular localization of StBBX14 in tobacco protoplasts.

BBXs play important roles in the plant response to cold stress [14,15]. Transcriptome analysis found that *StBBX14* was differently expressed under cold stress in *S. commersonii* (cold resistance) and *S. cardiophyllum* (cold sensitive). To investigate the *StBBX14* gene response to cold stress in potato, the gene expression level of *StBBX14* was examined by qRT-PCR. The results show that the expression level of *StBBX14* was significantly increased in 1 h and then gradually decreased under cold stress (Figure 1A). The coding sequence of *StBBX14* contains 1209 bp and encodes a protein of 402 amino acids. Phylogenetic analysis found that the *StBBX14* had the highest homology to the *SlBBX14* from *Solanum lycopersicum* L. (Figure 1B). To investigate the subcellular location of the StBBX14 protein, the CDS of *StBBX14* was cloned into the pAN580 vector under the CaMV35S promoter to construct an in-frame fusion protein plasmid 35S::pAN580-BBX14-GFP. This result suggests that the *StBBX14* protein is mainly localized in the nucleus of tobacco (Figure 1C).

### 2.2. Overexpression of StBBX14 in Potato Enhanced Cold Resistance

**Figure 2:**
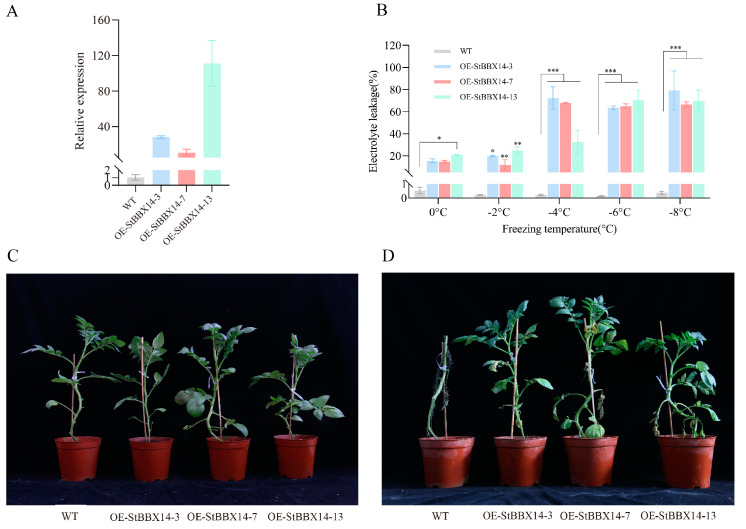
Overexpression of *StBBX14* increased the cold resistance. (**A**) The gene expressions of *StBBX14* in CK, *OE-StBBX14-3*, *OE-StBBX14-7*, and *OE-StBBX14-13* plants. (**B**) The electrolyte leakages of genotypes at the indicated temperatures. (**C**) Representative plants of the CK and *StBBX14* overexpression transgenic lines were photographed before exposure to the freezing treatment and after being subjected to −2 °C for 4 h (**D**), followed by 3 days of recovery under normal conditions. Student’s *t*-test was performed (* represented *p* < 0.05, ** represented *p* < 0.01 and *** represented *p* < 0.001). Each error bar represents the standard error (SE) calculated from three replicates. Means denoted by the same letter were not significantly different at *p* < 0.05 based on Student’s test.

To investigate whether *StBBX14* had a role in the regulation of cold stress, the CDS of *StBBX14* was placed under the control of the cauliflower mosaic virus CaMV 35S promoter, and the construct was introduced into potato for the generation of stable *StBBX14*-OE potato lines. The transcript levels of *StBBX14* OE-3, OE-7, and OE-17 plants were over 30 times higher when compared with the wild type (WT) (Figure 2A). The cold resistance of 30-day-old transgenic lines and WT grown in soil pots was used to assess *StBBX14*’s role in potato cold tolerance. The electrolyte leakage experiment results displayed that the three transgenic lines showed significantly lower electrolyte leakages at −2 °C, −4 °C, and −6 °C compared with the WT (Figure 2B). The transgenic lines and the WT displayed no obvious morphological differences under optimal growth conditions. Three overexpression lines, along with WT plants, were subjected to −2 °C for 4 h and recovery for 3 days without cold acclimation. The wild type (WT) suffered from more severe plant damage than the three transgenic lines. The three transgenic plants had some wilted leaves (Figure 2C,D). The electrolyte leakage results were in agreement with the observed phenotype. These results suggest that StBBX14 positively regulates the cold resistance in potato.

### 2.3. StBBX14 Activated the Expression of Multiple Cold-Responsive Gene

**Figure 3:**
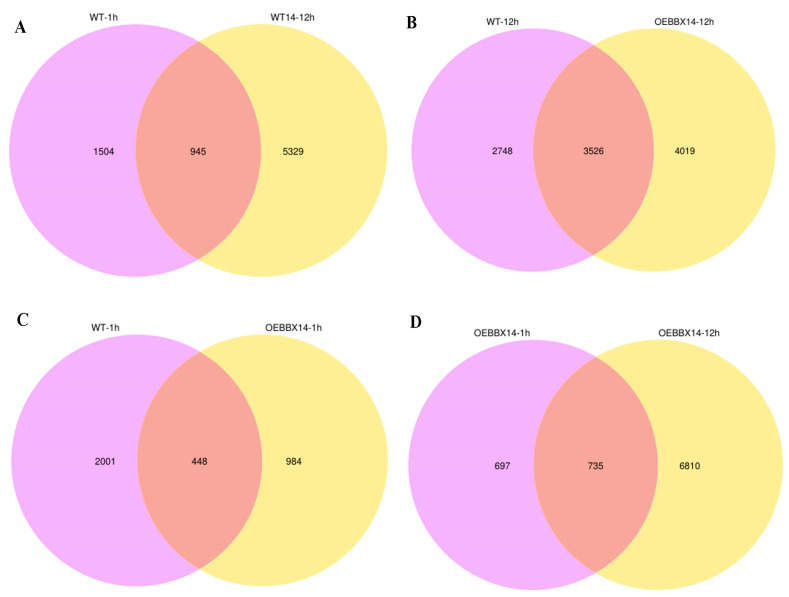
The numbers of DEGs in WT-1h vs. WT-12h (**A**), WT-12h vs. *OEBBX14*-12h (**B**), WT-1h vs. *OEBBX14*-1h (**C**), and *OEBBX14*-1h vs. *OEBBX14*-12h (**D**).

To investigate the role of *StBBX14* in regulating freezing tolerance, RNA-seq was conducted on *OE-StBBX14* and WT under normal and low-temperature conditions (2 °C for 0, 1, and 12 h). A total of 798 million raw reads were generated in eighteen libraries. After filtering out the low-quality reads and adapter sequences, 756 million clean reads were obtained (Table S1). The clean reads were mapped to the potato reference genome and the mapping rate ranged from 79.1–82.4%. Compared with WT-0h, totals of 2449 (Table S2) and 6274 (Table S3) differentially expressed genes (DEGs) were identified in WT-1h and WT-12h, respectively. Totals of 1432 (Table S4) and 7545 (Table S5) DEGs were detected in *OEBBX14*-1h and *OEBBX14*-12h when compared with *OEBBX14*-0h, respectively (Figure 3C,D). It was showed that more specific DEGs were detected after 12 h for either the WT or the *OEStBBX14*. In all, 945 and 3526 overlapping DEGs were identified in WT-1h versus vs. WT-12h and WT-12h vs. *OEBBX14*-12h (Figure 3A,B). Totals of 448 and 735 overlapping DEGs were obtained in WT-1h vs. *OEBBX14*-1h and *OEBBX14*-1h vs. *OEBBX14*-12h (Figure 3C,D).

The Gene Ontology (GO) enrichment analysis revealed that photosynthesis, cell wall, thylakoid, transcription regulator activity, oxidoreductase activity, and glucosyltransferase activity were significantly enriched in the DEGs of both OEBBX14 and the WT. The GO terms related to the peptide metabolic process and calmodulin binding were significantly enriched in OEBBX14. The cellular carbohydrate metabolic process and FAD binding were significantly enriched in WT. The Kyoto Encyclopedia of Genes and Genomes (KEGG) pathway enrichment analysis revealed that the KEGG pathways and photosynthesis and plant hormone signal transduction were significantly enriched in the DEGs of both OEBBX14 and the WT. The MAPK signaling pathway was significantly enriched in OEBBX14.

### 2.4. WGCNA Analysis

**Figure 4:**
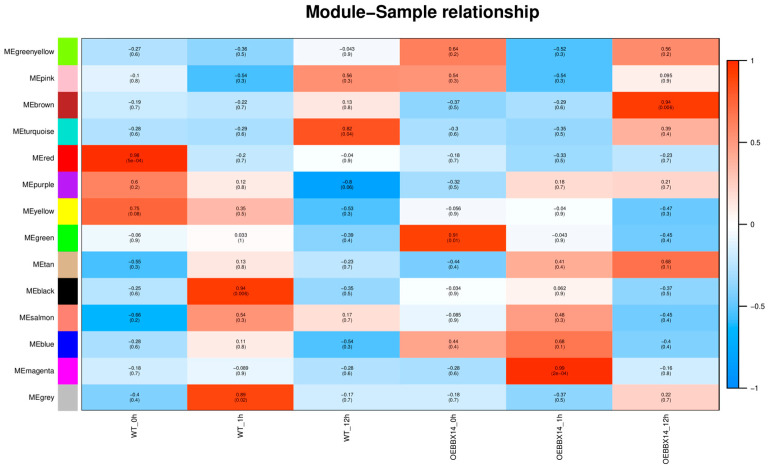
Module–sample relationships in potato under cold stress by weighted gene co-expression network. Each row denotes a module eigengene gene. Each cell comprises the corresponding correlation and *p*-value.

To identify the key genes associated with cold stress in potato, a WGCNA analysis was performed to construct the co-expression networks based for all the DEGs in OEBBX14 and the WT. A total of 14 distinct modules were generated (Figure 4). In the WT, the red module (*r* = 0.98, *p* = 5 × 10^−4^) was significantly correlated with WT-0h. In the OEBBX14, the green (*r* = 0.91, *p* = 1 × 10^−2^), magenta (*r* = 0.99, *p* = 2 × 10^−4^), and brown (*r* = 0.94, *p* = 6 × 10^−3^) modules were highly associated with OEBBX14-0h, OEBBX14-1h, and OEBBX14-12h, respectively.

**Figure 5:**
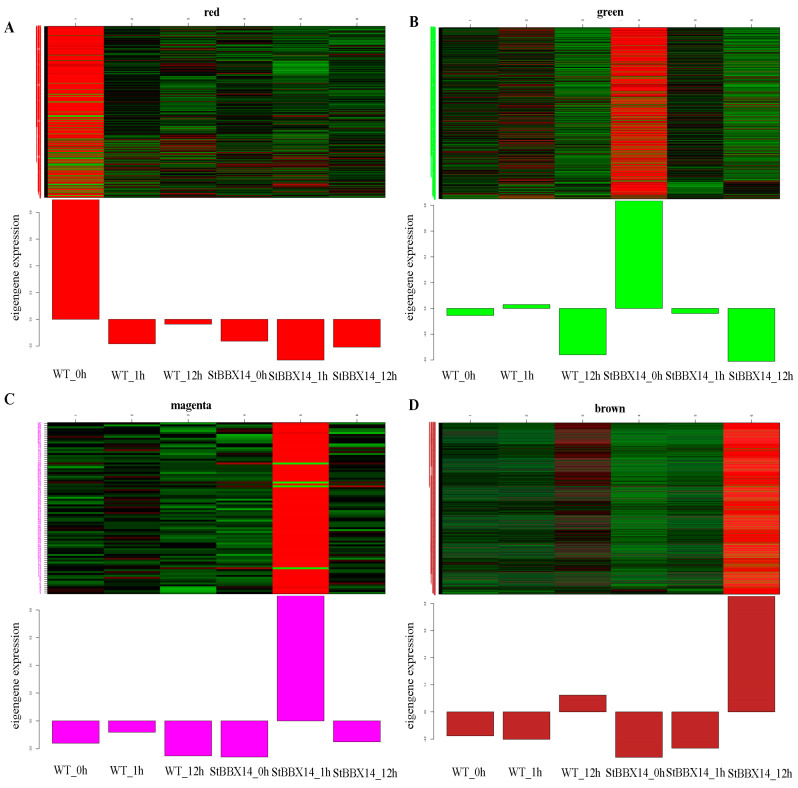
Gene expression patterns of four modules: (**A**) red module, (**B**) green module, (**C**) magenta module, and (**D**) brown module. The bar graph of eigengene expression displays the eigengene value calculated from the singular value composition for each module.

In the red module (477 genes), the putative functions of these DEGs were enriched in the GO categories ‘transcription regulator activity’, ‘DNA-binding transcription factor activity’, and ‘lyase activity’ (Figure 5A, Table S6). The DEGs in the red module were highly expressed in WT-0h, including *Stphytochrome interacting factor 5* (*StPIF5* K_ME_ = 0.99), *ABSCISIC ACID INSENSITIVE 5* (*StABI5*, K_ME_, 0.9), *mitogen-activated protein kinase kinase 4* (*StMAPKK4*, K_ME_, 0.94), *ethylene response factor 3 like* (*StERF3-like*, K_ME_, 0.9), and *ethylene response factor 60* (*StERF60*, K_ME_, 0.88). The ‘green’ module comprised 771 DEGs that were highly expressed in *OEBBX14*-0h (Figure 5B, Table S7), including five ethylene responsive element binding factor, *heat shock factor 24* (*StHSF24*, K_ME,_ 0.95), and *dehydration response element B1A* (*StCBF3*, K_ME_, 0.75). The GO terms related to the ‘DNA-binding transcription factor activity’ and ‘transcription regulator activity’ were the most enriched. The GO terms associated with ‘response to auxin’ and ‘response to endogenous stimulus’ were significantly enriched in the magenta module. All 90 DEGs in the magenta module were highly expressed in the samples of *OEBBX14*-1h, where 19 BRI1-associated receptor kinase, *glutathione S-transferase F11* (StGST1, K_ME_, 0.96), and glutathione S-transferase (*StGST1-like*, K_ME_, 0.8) were highlighted (Figure 5C, Table S8). In the brown module (1596 DEGs), the genes were significantly enriched in ‘nucleoside-triphosphatase activity’ and ‘ADP binding’. *ETHYLENE-INSENSITIVE3-like* (*StEIL1*, K_ME_, 0.96), *dehydration response element B1A* (*StCBF2*, K_ME_, 0.98), *xyloglucan endotransglycosylase 23* (*StXTH23*, K_ME_, 0.86), B-box type zinc finger family protein 4 (*StBBX4*, K_ME_, 0.95), and a total of nine MAPK genes in the brown module were highly expressed in the samples of OEBBX14-12h (Figure 5D, Table S9).

### 2.5. RT-qPCR Validation of Gene Expression

**Figure 6:**
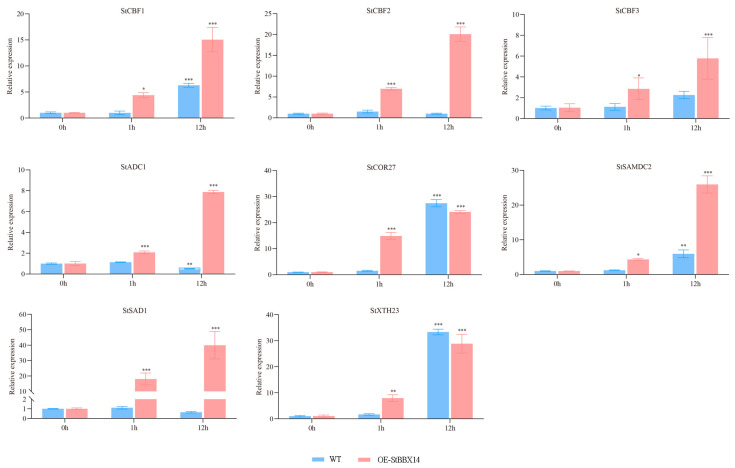
The expression levels of eight DEGs at 0 h, 1 h, and 12 h after the 2 °C cold treatment in the WT and *OE-StBBX14*. Student’s *t*-test test with * represented *p* < 0.05, ** represented *p* < 0.01 and *** represented *p* < 0.001 were used to indicate significant differences.

To validate the hub gene expression patterns in response to cold stress, eight DEGs were selected for RT-qPCR analysis (Figure 6). Compared with the WT, the *StADC1*, *StCBF1*, *StCBF2*, *StCBF3*, *StSAD1*, and *StSAMDC2* genes were significantly increased in *OE-StBBX14*-1h and *OE-StBBX14*-12h. Compared with the WT, the *StCOR27* and *StXHT23* genes were significantly increased in *OE-StBBX14*-1h and decreased in *OE-StBBX14*-12h. The expression patterns of the eight DEGs were consistent between the qPCR and RNA-seq, indicating the reliability of the transcriptome sequencing results.

## 3. Discussion

With global warming, extreme temperatures have become more prevalent. As an important abiotic stress, low-temperature stress was proved to affect the growth and development of crops. Plants adapt to low-temperature stress through changes in their morphology, physiology, biochemistry, and metabolic regulation [20]. Potato cultivars are not resistant to low-temperature frost, so it is important to explore the cold resistance gene of *S*. *commersonii*, namely, *ScSAD*, transformed in the cultivated potato variety Zhongshu 8 to significantly enhance the freezing tolerance of Zhongshu 8 [18]. Previous results showed that the *SaADC1* gene functioned in the cold-acclimated freezing tolerance of potato by promoting putrescine accumulation [19]. *SaCBL1-like* was exhibited to confer freezing tolerance via the *CBF* regulon in potato [17]. The overexpression of *ScAREB4* promotes freezing tolerance and functions as a downstream transcription factor of ABA signaling [21]. Although some genes related to potato cold resistance were functionally verified, the molecular mechanism of potato cold resistance has not been fully understood.

The BBX gene was demonstrated to play a pivotal role in plant cold tolerance [14,15]. In Arabidopsis, a total of 32 BBX family genes were identified [22]. Previous research indicated that the *CRYPTOCHROME2* (*CRY2*)-*COP1*-*HY5*-*BBX7/8* module plays important roles in the regulation of blue-light-dependent cold acclimation in Arabidopsis [15]. *MdBBX37* bound to the *MdCBF1* and *MdCBF4* promoters to activate their transcription and MdBBX37 cold resistance in apple [7]. A 27 bp InDel in the promoter of the *SlBBX31* is significantly associated with cold tolerance in tomato [16]. Although 30 BBX family genes were detected in potato [23], it is still necessary to further explore the gene function of the *BBX* gene family in potato. In this study, overexpression of the *StBBX14* gene displayed less leaf damage and significantly lower electrolyte leakage compared with the WT under cold stress, indicating that *StBBX14* increased the potato’s cold resistance capabilities. Previous studies suggested that the CBFs play key roles during cold acclimation in plants [24,25]. Ectopic *AtCBF1* overexpression exhibited improved cold acclimation in transgenic *S. commersonii* [26]. When comparing the ectopic overexpression of *StCBF1* and *ScCBF1* in *Arabidopsis*, *ScCBF1* showed much more tolerance to freezing than *StCBF1* [27]. In this study, the expression levels of StCBF1, *StCBF2*, and *StCBF3* were significantly increased in *OE-StBBX14*-1h and *OE-StBBX14*-12h. The overexpression of *SlBBX17* positively regulates cold tolerance through a CBF-dependent process in tomato [14]. It was found that *BBX29* is a negative regulator of cold stress by ABA- and CBF-independent pathways in Arabidopsis [28]. Taken together, the *BBX* gene can regulate low temperature expression depending on a *CBF*-independent or -dependent pathway in plants.

The RNA-seq technique was employed to compare gene expression profiles between *OE--StBBX14* and the WT under cold stress. The DEGs were detected in 1 h and 12 h in either the WT or *OE-StBBX14*. A total of 14 distinct modules were generated by a WGCNA analysis. Five modules were highly correlated with the cold stress. In the red module, *StPIF5*, *StABI5*, and *StMAPKK4* were highly expressed in WT-0h. The results imply that *PIF1*, *PIF4*, and *PIF5* negatively regulate the cold stress in *Arabidopsis* [29]. *MaABI5* in the ABA signaling pathway is involved in cold tolerance by interaction with *MaC3HC4-1* in banana [30]. Compared with the wild type, the overexpression of *SaMKK2* was increased the expression of CBF1/2/3 under cold stress [31]. The cytochrome P450, MYB, and WRKY gene family were highly expressed in WT-1h. The transcription factors play key roles in plant responses to cold stresses. The apple *MdMYB308L* positively regulated cold tolerance by binding to the promoters of *MdCBF2* [32]. A total of 143 differentially expressed cytochrome P450 genes were identified under cold stress in tomato [33]. The overexpression of *VaWRKY3*3 enhanced the cold tolerance in grape calli with lower low-temperature exothermic values than the empty vector calli [34]. *StCBF3* and *StHSF24* were highly expressed in *OE-StBBX14*-0h in the ‘green’ module. In *S. commersonii*, all four *CBF* genes were cold responsive, with *CBF1* and *CBF3* being the most actively responsive under both acclimated and nonacclimated conditions [35]. The *PpHSFA4c*-mediated *HSF*-*HSP* and ROS pathways promote fruit cold resistance in peach [36]. The *StGST1* and *StGST1-like* were highly expressed in the samples of *OE-StBBX14*-1h in the magenta module. The *BoGSTU19*, *BoGSTU24*, and *BoGSTF1* genes were highly expressed at 6 h and 1 h in the cold-tolerant and cold-susceptible lines of *B. oleracea* [37]. In the brown module, *StEIL1*, *StCBF2*, *StXTH23*, *StBBX4*, and a total of nine *MAPK* genes in the brown module were highly expressed in the samples of *OE-StBBX14*-12h. In upland cotton, *XTH22* positively regulated the response to cold stress [38]. These results provide abundant data to explore the molecular mechanism of potato’s response to cold stress.

## 4. Materials and Methods

### 4.1. Materials and Growth Conditions

The low-temperature-sensitive potato cultivar ’Desiree’ was used in this study. The Désirée variety of potato has light yellow flesh and light red skin. The tubers of the Désirée variety are medium-large in size, oblong in shape, with shallow eyes. They have red skin and yellow flesh. The plants are tall and erect, the leaves flat, slightly divided. Many reddish-purple flowers appear in summer and form fruits. Désirée is a semi-late to late variety. It is then harvested 145 days after sowing, when the foliage has completely withered. Initially, the cultivar was inoculated on an MS medium supplemented with 3% sucrose and 0.7% agar and cultured in an incubator maintained at 22 ± 2 °C, with a light intensity of 2400 Lux and a photoperiod of 16 h/8 h. After four weeks of in vitro culture, the plants were transferred to the field. The potato tubers were harvested 12 weeks post-transplantation for subsequent analyses.

### 4.2. Sequence and Phylogenetic Analysis of StBBX14

The amino acid sequences of BBX family proteins from various plant species were retrieved from monocot and dicot libraries, including the *StBBX14* gene (ID: Soltu.DM.03G034030.1) from the potato genome database. Sequence alignments were performed using DNAMAN 6.0 software. Phylogenetic trees were constructed using the maximum likelihood method with MEGA 7.0 software, set to 1000 bootstrap replicates to assess the reliability of the inferred phylogeny.

### 4.3. Subcellular Localization Analysis

The coding sequence of StBBX14 was PCR-amplified and cloned into the pAN580-linker vector and tagged with GFP. This construct was transformed into tobacco protoplasts, and GFP fluorescence was observed under a Nikon C2-ER laser confocal microscope (Nikon, Tokyo, Japan) using an excitation wavelength of 488 nm [39].

### 4.4. Frost Resistance Determination

To assess the frost tolerance of the transgenic plants, the electrolyte leakage from the leaves was measured. Relative leaves of the second and third compound leaves were taken, and the assay method was described in a previous paper [18]. The ion leakage was expressed as the ratio of electrolyte leakage from frostbitten tissue (R1) to the electrolyte leakage after autoclaving (R2). The relative electrolyte leakage rate was calculated using R1/R2 × 100%. In addition, LT50 (half-lethal temperature) was calculated based on the electrolyte leakage to measure the freezing resistance.

### 4.5. RT-qPCR Analysis

We extracted the total RNA using the Plant Total RNA Kit (OMEGA, Norcross, GA, USA). First-strand cDNA was synthesized using the Reverse Transcription Kit TRUEscript RT MasterMix (XinBaiJi Biotech, Nanjing, China). First-strand cDNA was synthesized using the BlasTaqTM 2X qPCR MasterMix (Abm, Vancouver, BC, Canada) on a Bio-RadCFX96machine (Bio-Rad Laboratories, Hercules, CA, USA) for RT-qPCR. Calibration and standardization were performed with the internal reference base β-tubulin2. Three replicates were set up for each biological sample, and the relative expression of genes was calculated using the 2^−ΔΔCt^ method.

### 4.6. Plasmid Construction and Genetic Transformation

Homologous recombination was used to ligate the vector pFGC1008 linearized with Kpn I and Sac I. The overexpression vector plasmid was constructed and subsequently transformed into Agrobacterium rhizogenes GV3101 by the heat-shock method. The low-temperature-sensitive variety ‘Desiree’ was used for the Agrobacterium-mediated transformation. Genetic transformation of potato miniature tubers followed the method described in a previous paper [18]. To test the effectiveness of the transgenic plants, PCR amplification with the correct size was used to identify positive plants. qRT-PCR was used to further detect the gene expression of *StBBX14* in the transgenic lines. The transgenic potatoes were planted in the field, and the WT plants were cultured under the same conditions as a control. After 30 days, they were subjected to low-temperature treatment at −2 °C for 4 h for phenotypic characterization. The primers used for amplification and qRT-qPCR are provided in Table S10.

### 4.7. Transcriptome Analysis

The 4-week-old wild-type (WT) potato and StBBX14 overexpression lines were treated with a 2 °C cold treatment for 0 h, 1 h, and 12 h. Each treatment with 3 biological replicates underwent RNA sequencing. Reads with splice adapters, unidentifiable base information, and low-quality reads were removed and filtered so that clean reads from each sample were aligned to the potato reference genome. The FPKMs (Fragments Per Kilobase of transcript Per Million Fragments) indicate the number of fragments per kilobase per million reads, where the sequencing depth and gene length were corrected according to the FPKMs [40]. HISAT2 v2.0.5 software was used to analyze the differentially expressed genes (DEGs) in the wild-type and transgenic plants under the low-temperature treatment. FDR ≤ 0.05 and log2|(FoldChange)| ≥ 1 were used as thresholds for significant differential gene expression. The differential gene sets were analyzed by clusterProfiler (3.8.1) software for the GO function enrichment analysis and KEGG pathway enrichment analysis to determine the biological functions and pathways.

### 4.8. Weighted Correlation Network Analysis (WGCNA)

The outlier samples were filtered by expression matrix correlation, and the gene co-expression network was constructed by the R package WGCNA 4.0.3 [41]. The automatic network building function was employed to acquire co-representation modules, and the correlation between modules and processing was calculated to obtain the eigenvalues of each module. 

### 4.9. Statistical Analysis

The data were subjected to statistical analysis using Student’s *t*-tests or one-way analysis of variance, and all graphs were generated using the Prism 9.5 software package. The results are presented as the mean ± standard deviation. Significant differences between means were indicated by asterisks (* *p* < 0.05, ** *p* < 0.01 and *** *p* < 0.001).

## 5. Conclusions

In this study, the overexpression of StBBX14 in potato enhanced the cold resistance. The Gene Ontology (GO) enrichment analysis revealed that photosynthesis, cell wall, thylakoid, transcription regulator activity, oxidoreductase activity, and glucosyltransferase activity were significantly enriched in the DEGs of both OEBBX14 and WT. A total of 14 distinct modules were generated by a WGCNA analysis. Four major modules with cold-related genes were isolated. RT-qPCR analysis showed that the expression patterns of eight DEGs were consistent between the qPCR and RNA-seq. These findings illustrate that the StBBX14 plays an important role in cold stress in potato and provides a data basis for the genetic improvement of cold resistance traits of potato.

## Data Availability

The data generated in this study can be found in this article.

## Supplementary Materials

The following supporting information can be downloaded from https://www.mdpi.com/article/10.3390/plants14010018/s1. Supplementary Table S1. The RNA-seq information in WT and OE-BBX14. Supplementary Table S2. The DGEs information in WT_1h. Supplementary Table S3. The DGEs information in WT_12h. Supplementary Table S4. The DGEs information in OE-BBX14_1h. Supplementary Table S5. The DGEs information in OE-BBX14_12h. Supplementary Table S6. The genes information in red module. Supplementary Table S7. The genes information in green module. Supplementary Table S8. The genes information in magenta module. Supplementary Table S9. The genes information in brown module. Supplementary Table S10. Primer sequences used in this study.
